# APPLICATIONS OF QUALITATIVE RESEARCH IN DESCRIBING PCC ENVIRONMENTS: THE STORY BEHIND THE STATISTIC

**DOI:** 10.1093/geroni/igz038.2053

**Published:** 2019-11-08

**Authors:** Migette Kaup, Judith Poey, Gayle Doll, Laci Cornelison

**Affiliations:** 1 Kansas State University, Manhattan, Kansas, United States; 2 United Way of Central Maryland, Baltimore, Maryland, United States

## Abstract

The goal of PCC is to enhance quality and bring meaning to the lived experience in long-term care. This requires attention to patterns of life, and the creation of residential experiences within the environment. Ten case studies of homes participating in a pay-for-performance PCC program in the Midwest reveals how environmental affordances may be critical in the implementation and sustainability of PCC. Data collected through an in-depth environmental assessment revealed multiple strategies and attributes related to organizational, operational, and environmental practices. This presentation will demonstrate a diagrammatic and empirical comparison of the environments of those early in the process and those who have fully implemented and sustained PCC practices. Specific features will be highlighted and patterns discussed.

